# Security, Privacy and Risks Within Smart Cities: Literature Review and Development of a Smart City Interaction Framework

**DOI:** 10.1007/s10796-020-10044-1

**Published:** 2020-07-21

**Authors:** Elvira Ismagilova, Laurie Hughes, Nripendra P. Rana, Yogesh K. Dwivedi

**Affiliations:** 1 School of Management, University of Bradford, Richmond Road, Bradford, BD7 1DP, UK; 2 Emerging Markets Research Centre (EMaRC), School of Management, Swansea University, Bay Campus, Fabian Way, SA1 8EN Swansea, UK

**Keywords:** Smart cities, Literature review, Privacy, Security, Risk, Interaction framework

## Abstract

The complex and interdependent nature of smart cities raises significant political, technical, and socioeconomic challenges for designers, integrators and organisations involved in administrating these new entities. An increasing number of studies focus on the security, privacy and risks within smart cities, highlighting the threats relating to information security and challenges for smart city infrastructure in the management and processing of personal data. This study analyses many of these challenges, offers a valuable synthesis of the relevant key literature, and develops a smart city interaction framework. The study is organised around a number of key themes within smart cities research: privacy and security of mobile devices and services; smart city infrastructure, power systems, healthcare, frameworks, algorithms and protocols to improve security and privacy, operational threats for smart cities, use and adoption of smart services by citizens, use of blockchain and use of social media. This comprehensive review provides a useful perspective on many of the key issues and offers key direction for future studies. The findings of this study can provide an informative research framework and reference point for academics and practitioners.

## Introduction

The term ‘smart cities’ generally refers to the use of technology-based solutions to enhance the quality of life for citizens, improve interaction with government and promote sustainable development (Chourabi et al. 2012; Yahia et al. 2019; Yu and Xu 2018). A city can be described as smart where social, environmental and economic development factors are balanced and linked via devolved processes to more efficiently manage key assets, resources and urban flows for real-time processes (Komninos 2013; Yeh 2017). Smart cities are designed around an Information and Communication Technology (ICT) based infrastructure with Internet of Things (IoT) enabled sensor technology to support social and urban interconnectivity through greater citizen interaction and government efficiency (Albino et al. 2015; Alter 2019; Gupta et al. 2019b; Janssen et al. 2019; Lom and Pribyl 2020; Mamonov and Koufaris 2020; Manfreda et al. 2019; Yeh 2017). A number of cities throughout the world have embraced the smart philosophy and have either developed their infrastructure toward this new status or are actively pursuing strategies to adapt their existing assets and networks. These include London, New York, Paris, Amsterdam, Reykjavik, Tokyo, Busan, Dubai, Stockholm and Santander (Forbes 2019; Peris-Ortiz et al. 2016; Simonofski et al. 2019). The Government of India (GoI) has announced plans to develop 100 smart cities throughout the country to drive economic growth via the creation of technological solutions for citizen interaction (Praharaj et al. 2018). The top down, government led approach within China has led to a large number of smart city projects that are viewed as policy based decisions to potentially reshape economic structures, transform economic development, re-educate and enhance the competitiveness of workers and improve government capacity and efficiency in the context of energy management and environmental pollution (Yu and Xu 2018). The technology spending on smart city initiatives worldwide is currently $81 billion and predicted to reach $158 billion (US) by 2022, highlighting the significance attached to these city transformations (Statista 2019).

Smart city designers utilise modern technologies such as mobile cloud computing, electronic objects, networks, sensors and machine learning technologies to enable the different components of smart cities to cooperate and interact with the network architecture (Ismagilova et al. 2019b; Singh et al. 2019). The inherent complexity and new methods of citizen interaction required for the change to existing infrastructure, highlights the significant political, regulatory and technical challenges for governments and regional authorities. One of the key challenges in the development of smart cities is the processing and management of data. This relates to data already present within city databases but also the linking of data with new systems and sensors present within the smart city, that impact security and privacy (Van Zoonen 2016). The threats stemming from information security, data privacy and cyber-related factors where unauthorised access to information can cause undesired consequences, highlights the criticality of addressing these issues early within the design and development stage of smart cities (Chatterjee et al. 2019; Elmaghraby and Losavio 2014).

The literature has developed a number of studies that have presented reviews of the smart city literature (Albino et al. 2015; Anthopoulos 2015; Bibri and Krogstie 2017; Chatterjee and Kar 2015; Ismagiloiva et al. 2019a; Kar et al. 2019; Rana et al. 2019). However, researchers seem to have omitted to offer a meaningful analysis of the significant threats to data security and the inherent complexities surrounding privacy within smart cities. This research aims to bridge this gap in the literature by conducting a comprehensive analysis of the many issues and key complexities relating to privacy, security, and risk issues within smart cities. In alignment with the guidance in Pare et al. (2015), we conduct a theoretical review of current studies and available literature on privacy, security, and risks within smart cities and develop a smart cities security & privacy framework.

The current study aims to address the following questions:

- What is the current state of knowledge relating to security, privacy, and risk within smart cities?
- What are the smart cities’ challenges in areas relating to privacy, security, and risk from a number of stakeholder perspectives?

The analysis and findings of this research are presented as offering an informative and timely framework for research on this topic, useful to academics and practitioners alike.

The remaining sections of the paper are organised as follows. Section 2 provides an overview of the methods used to identify relevant studies to be included in this research. Section 3 details the discussion of the emerging themes from the existing research on security and privacy in smart cities. Section 4 discusses many of the key aspects of the research in the context of significant challenges to the further development of smart cities and presents a conceptual framework on security and privacy issues. The study is concluded in Section 5. The limitations and directions for future research are presented in Section 6.

## Research Methodology

The approach utilised in this study aligns with the recommendations in Moher et al. (2009) and Kitchenham (2004). The study applied the following steps for systematic search of the relevant papers: (1) development of protocol; (2) filtration of research papers by title, keywords and abstract; (3) extraction of data from the selected papers.

*Development of protocol.* To identify relevant articles from the literature a keyword search was employed. The search was restricted to peer-reviewed research papers. A number of separate keyword searches were utilised, namely “Smart City” OR “Smart Cities” AND “Privacy” OR “Security” OR “Risk” were searched via Scopus database. The Scopus database was selected for this study as it includes a catalogue of more than 50 million records from approximately 20,500 titles and 5,000 publishers (Montoya et al. 2016). As a result, this database can be useful for searching and locating a substantial proportion of the published peer-reviewed research papers in the area of smart cities research. Additionally, by using online databases for conducting a systematic literature the search aligned with the emerging culture narrative, highlighted by a number of information systems researchers (Gupta et al. 2019a; Heidt et al. 2019; Hughes et al. 2019; Papagiannidis and Marikyan 2020; Tamilmani et al. 2020). From the Scopus database, only peer-reviews papers that were published in English were considered. The search resulted in 99 articles returned. This comprehensive search strategy allowed us to minimise source bias and return sufficient articles for a comprehensive review (Dwivedi et al. 2019).

### Filtration of research papers by title, keywords and abstract

All studies were analysed and processed by the authors then reviewed based on title, keywords and abstract to ensure validity and relevance that the research offered contribution to the smart cities’ discussion in the context of risk, privacy and security. Based on the inclusion criteria, the review of the 99 articles yielded 94 that were selected as offering relevance for this study.

### Extraction of data from the selected papers

The following data were extracted from each research paper: (1) Year of publication of the research paper; (2) Name of the journal in which the research paper was published; (3) Bibliographic reference including title, year, author, and source of the research paper; (4) The main objective of the research paper; and (5) Findings of the research paper. The selected articles have appeared in 30 separate journals, including seven journals that have published two or more articles relating to privacy and security in smart cities (Table 1). The remaining 23 journals have contributed just one article each. To systematically provide academic insights on the research themes, identified studies were divided into broad smart cities related key themes.

**Table 1:** Journals publishing two or more studies on security, privacy and risk within smart cities

Journal	No of studies
Personal and Ubiquitous Computing	4
Journal of Information Science and Engineering	3
Computers and Security	3
International Journal of Advanced Computer Science and Applications	3
Computer	2
Future Generation Computer Systems	2
Government Information Quarterly	2
Other	76

**Fig. 1:**
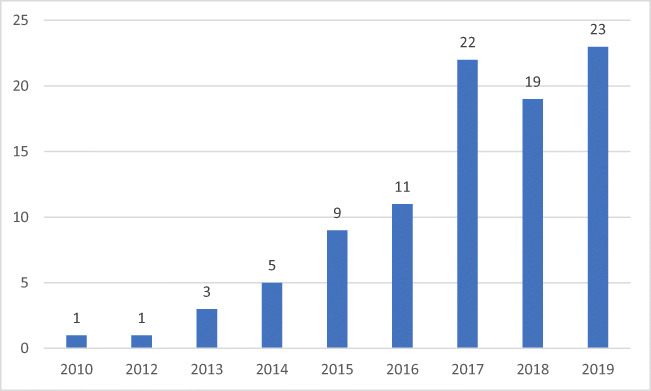
Publications on privacy, security, and risks in smart cities: 2010–2019

The chronological view by volume of the articles on security and privacy within smart cities is presented in Fig. 1 and depicts the increasing academic interest over recent years.

## Literature Review

The wider literature has tended to focus on a number of broad smart cities related key themes namely: the privacy and security of mobile devices and services; smart cities infrastructure and technical architecture; power systems utilised within smart cities; smart healthcare; security and privacy frameworks; algorithms and protocols; operational threats for smart cities; application of blockchain solutions within smart cities; and social media and smart cities.

**Fig. 2:**
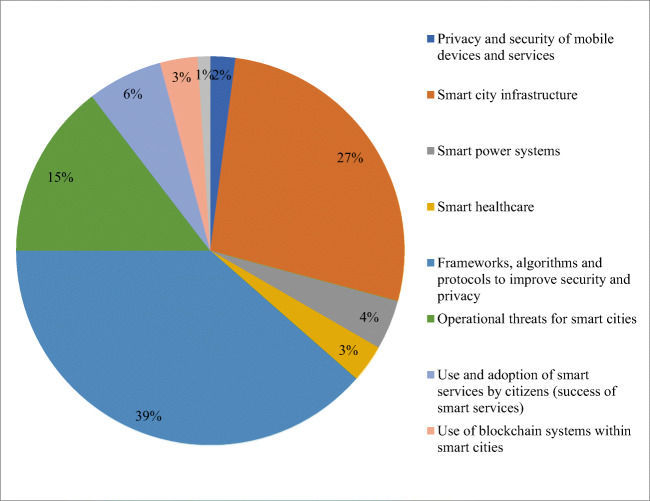
Clusters of research themes

Figure 2 presents the research themes and the percentage of papers grouped under each theme. The themes and related references are listed in Table 2. The subsections of the article generated discussion around each theme providing insights to research carried under the respective main themes

**Table 2:** Smart cities themes and references within the literature

Theme	References
Privacy and security of mobile devices and services	Abi Sen et al. (2018); Li et al. (2019)
Smart city infrastructure	Abosaq (2019); Ainane et al. (2018); Alandjani (2018); Antoine Picon (2019); Awad et al. (2019); Baryshev et al. (2016); Bernardes et al. (2018); Chatterjee et al. (2017); de Amorim et al. (2019); de Fuentes et al. (2017); Efthymiopoulos (2015); Elmaghraby and Losavio (2014); Evans (2018); Ferraz and Ferraz (2014a); Hiller and Blanke (2017); Jameel et al. (2019); Khan et al. (2014); Li et al. (2015); Liao et al. (2017); Liu et al. (2017); Pérez-Martínez et al. (2013); Rohokale and Prasad (2017); Vitunskaite et al. (2019); Waedt et al. (2016); Wang et al. (2012); Zhu and Zuo (2015)
Smart power systems	Ainane et al. (2018); Alamaniotis et al. (2017); Karasevich et al. (2016); Sanduleac et al. (2016)
Smart healthcare	Alromaihi et al. (2018); Huang et al. (2017); De Fuentes et al. (2018)
Frameworks, algorithms and protocols to improve security and privacy	Al-Dhubhani et al. (2018); Antonopoulos et al. (2017); Avgerou et al. (2016); Beltran et al. (2017); Burange and Misalkar (2015); Cagliero et al. (2015); de Fuentes et al. (2017); Ferdowsi et al. (2017); Gheisariy et al. (2019); González García et al. (2017); Gope et al. (2018); Guo et al. (2017); Han et al. (2019); Huerta and Salazar (2019) ; Khedr et al. (2019); Krichen and Alroobaea (2019); Krichen et al. (2018); Lai et al. (2017); Lepinski et al. (2016); Luo et al. (2017); Mazhelis et al. (2016); Patsakis et al. (2015); Peters et al. (2019); Roldan et al. (2019); Sen et al. (2013); Shen et al. (2017); Song et al. (2017); Stromire and Potoczny-Jones (2018); Sucasas et al. (2018); Sucasas et al. (2016); Berg et al. (2019); Wibowo (2018); Witti and Konstantas (2018); Xiao et al. (2017); Xie and Hwang (2019); Yilei and Leyou (2019); Zang et al. (2017)
Operational threats for smart cities	Aldairi and Tawalbeh (2017); Baig et al. (2017); Dewi Rosadi et al. (2018); Dhungana et al. (2015); Ferraz and Ferraz (2014b); Galdon-Clavell (2013); Grieman (2019); Habibzadeh et al. (2018); Kitchin and Dodge (2019); Pérez-Martínez et al. (2013); Techatassanasoontorn and Suo (2010); Vattapparamban et al. (2016); Velasquez et al. (2018); Yang and Xu (2018)
Use and adoption of smart services by citizens (success of smart services)	Babdullah et al. (2017); Belanche-Gracia et al. (2015); Chatterjee et al. (2018); Cilliers and Flowerday (2014); Cilliers and Flowerday (2015); Slade et al. (2013); Slade et al. (2014); Van Heek et al. (2016); van Zoonen (2016)
Use of blockchain systems within smart cities	Mora et al. (2019); Noh and Kwon (2019); Ramos and Silva (2019)
Social media and smart cities	Moustaka et al. (2019)

### Privacy and Security of Mobile Devices and Services

Mobile devices are the backbone of interacting with the smart cities network infrastructure but present new challenges to the security and privacy of users where sensitive data could be vulnerable to attack by third parties. The Abi Sen et al. (2018) study proposed the use of fog computing properties such as caching, cooperating and acting as a broker between users and use of the cloud to mitigate security threats. The study presented three novel approaches for satisfying the required privacy of mobile devices within smart cities. The first approach utilised the concept of foggy dummies to protect the privacy of the user; the second incorporated a blind third party where a trust relationship is developed to protect the user from the server provider; the third approach used the concept of a double foggy cache to solve the trust issue between peers with a traditional cooperation approach. The Abi Sen et al. research posits the advantages of these approaches where there is no requirement to trust the party fully. The authors assert there is less overhead when compared to private information retrieval and the server provider cannot collect data on behavioural aspects of the user.

Privacy-preserving authentication (PPA) protocols for mobile services have emerged as a promising cryptographic approach to provide authentication and privacy protection features for smart cities. The research presented in Li et al. (2019) analysed the PPA protocol suitability for mobile services within a typical mobile service application in a smart city context. The research findings outlined the efficiency of PPA when compared to other competing protocols, demonstrating that the proposed PPA protocol would exhibit less computation and communication overheads when deployed in mobile service applications for smart cities.

### Smart City Infrastructure

A number of articles focused on smart city infrastructure and ways to overcome security and privacy issues within smart cities (Abosaq 2019; Ainane et al. 2018; Alandjani 2018; Antoine Picon 2019; Awad et al. 2019; Baryshev et al. 2016; Bernardes et al. 2018; Chatterjee et al. 2017; de Amorim et al. 2019). The IoT plays a pivotal role within the infrastructure of smart cities as it provides the network architecture responsible for gathering and processing data from distributed sensors and smart devices. Studies generally categorise attacks on IoT devices into *external* and *internal* - attacks (Alromaihi et al. 2018; Mo et al. 2010).

The vulnerability of IoT based applications is directly related to the network paradigm where physical objects such as sensor based devices collect data on key interactions within the network and communicate via wireless or wired connections. The data which is uploaded, processed and stored can exhibit key vulnerabilities in the form of man-in-the-middle attacks and denial-of-service attacks. As a result, collecting and transferring data via the use of IoT infrastructure could severely impact the security and privacy of smart cities unless precautionary measures are implemented (Awad et al. 2019). Studies have argued that privacy can be easily compromised due to the high levels of interaction between people, devices and sensors, thus highlighting the need for this data to be fully protected (Antoine Picon 2019; Elmaghraby and Losavio 2014). Studies have posited the merits of a more strategic focus on smart city security looking beyond aspects of data privacy toward a smart securitisation policy (Efthymiopoulos 2015). The study by Ferraz and Ferraz (2014a) argued that information security does not only include privacy, confidentiality, integrity and availability, but also includes interoperable security that represents the idea of a general failure of the urban system.

The data flows and exchanges between network components and the IoT should be subject to effective risk management in assessing and responding to threats within smart cities and the challenges of the technical sophistication gap and standards immaturity (Ainane et al. 2018; Alandjani 2018). Researchers have sought to identify technological solutions to deal with privacy and wider information security challenges. The study by Abosaq (2019) analysed the privacy issues faced by smart cities including authentication, access control, confidentiality, trust, data security, policy implementation and secure middleware. The author designed and simulated a smart city model connected with mandatory communication devices that produced data for a number of sensors. The study proposed that data privacy can be achieved by a Fast ID Online (FIDO) authentication process (Fido Alliance 2019) for the *device to network* or *device to cloud* authentication and that data privacy should be considered an integral element of the smart city infrastructure (Abosaq 2019). The privacy aspects inherent within smart city network traffic infrastructure were analysed in De Fuentes et al. (2017), where the study posited the benefits of an Attribute-Based Credentials (ABCs) solution to help address the issue of disclosure of unnecessary data. The research recommended an Idemix based approach due to its performance efficiencies and compatibility with existing smart city road traffic services. The research by Hiller and Blanke (2017) posited the suitability of utilising resilience theory which is concerned with the ability of an organism to survive and evolve into better states. The study views privacy as a system and examines it through the resiliency lens, framing the question of how privacy can adapt and survive within a smart city.

Khan et al. (2014) identified a list of stakeholders and modelled their involvement within the smart city context. The stakeholder mapping included: service consumers, legitimate service providers, untrusted service providers, IT experts, data custodians, standard governing bodies and domain experts. Based on the proposed stakeholder model, the study developed a security and privacy framework for secure and privacy-aware service provisioning in smart cities. The framework aimed to provide end-to-end security and privacy features for trustable data acquisition, transmission, processing and legitimate service provisioning, demonstrating the proposed frameworks ability to mitigate stakeholder security and privacy concerns. Additional relevant frameworks include the one proposed in Vitunskaite et al. (2019), that performed a comparative smart city case study of Barcelona, Singapore and London on their governance models, security measures, technical standards and third party management. The framework encompassed technical standards, governance input, regulatory framework and compliance assurance to ensure information security is observed within all layers of the smart city infrastructure.

Smart cities are comprised of a significant number of different sensors, interaction devices, network access points, specialised hardware and software. These key assets need to be integrated within the smart city infrastructure and maintained to ensure systems are not degraded and valuable services are operable. The study by Waedt et al. (2016) focused on the manual and automatic asset identification, annotation and tracking of graded Application Security Controls (ASCs) that can benefit from comprehensive and formalized asset management. This included the availability and integrity of fixed and mobile technology assets and the reliability and integrity of software assets installed on servers and cloud environments. The Waedt et al. (2016) study asserts that rigorous and pervasive asset management provides value beyond security to mitigate the misuse of assets for sophisticated attacks targeting combinations of version-specific vulnerabilities.

### Smart Power Systems

The power system aspects of smart cities are of critical importance within the overall security and privacy infrastructure, as third parties connected to the grid could monitor usage patterns and predict consumers’ behaviour. The wireless network technology focussed nature of the many systems that supply and control heat and light to smart cities, could expose the grid to security vulnerabilities. Alamaniotis et al. (2017) presented an intelligent methodology for enhancing privacy within smart power systems. The proposed methodology utilised demand patterns for several consumers connected to the power grid to provide a new consumption pattern. The new pattern hides individual consumer characteristics via a particle swarm optimisation process. The study tested the proposed methodology on a set of real consumption patterns benchmarked against a genetic algorithm demonstrating that the proposed methodology is efficient. The study by Sanduleac et al. (2016) addressed two main aspects of smart city implementation; namely: (i) multi-energy streams when different utilities serve different energy networks in the city such as electricity, gas, and heat (ii) the issue of engaging the citizens by sharing their private energy data profile, as an alternative to implementations that fail to progress from small pilots to large deployment.

### Smart Healthcare

The security and privacy of healthcare services and concepts within smart cities are a key factor in the overall minimal disclosure of data and information security infrastructure (Maria De Fuentes et al. 2018). The research by Alromaihi et al. (2018) identified the main security and privacy challenges in designing IoT architecture in the context of healthcare applications, highlighting the increased use of sensors for medicine and healthcare applications over the last decade. The study identified key threats from personal health related data captured via sensors for e.g. heart rate and also blood pressure and the importance of an integrated security solution for the entire system. The benefits of data collaboration within a secure mobile healthcare and social system have been proposed in Huang et al. (2017). The study developed a solution that allowed a data owner to authorise third party healthcare provider data analysis by re-encrypting Attribute-Based Encryption (ABE) and identity-based broadcast encryption (IBBE). The proposed scheme used encryption and decryption processes that effectively delegate most of the computation cost to the cloud. As a result, the computation overhead of resource-constrained mobile devices are reduced, with subsequent improvements in security and efficiency.

### Frameworks, Models, Algorithms and Protocols to Improve Security and Privacy

As smart cities face a number of challenges connected to security and privacy, some studies proposed various frameworks, models and algorithms to improve these issues (Al-Dhubhani et al. 2018; Antonopoulos et al. 2017; Avgerou et al. 2016; Beltran et al. 2017; Burange and Misalkar 2015; Cagliero et al. 2015). This aspect of the literature has focused on encryption algorithms to build in security to smart city systems. The Antonopoulos et al. (2017) study tests high-level security feature algorithms by using Wireless Sensor Network (WSN) development. Stromire and Potoczny-Jones (2018) proposed to integrate an end-to-end cryptography system into smart city solutions at a foundation level. During any data breach, nothing about the data would be revealed by applying this system. Similarly, Lai et al. (2017) used an encryption approach in proposing a scheme titled Fully Privacy-Preserving and Revocable Identity-Based Broadcast Encryption (FPPRIB). The proposed scheme aimed to preserve the data privacy and the identity privacy of the receiver as well as the revoked user. The data can be securely protected and only the authorised user can access the data. The revocation process does not reveal any information about the data contents or the receiver identity and the public learn nothing about the receiver identity and the revoked user identity. These properties lead to applications in the smart city where identity privacy is desirable. The study by Patsakis et al. (2015) developed a cryptographic protocol which manages the huge amount of personal information that could be generated through e-participation in a scalable, interoperable manner, which guarantees the privacy of citizens within smart cities.

Network access control plays an important role in any communication system. It is important to develop adequate security of IoT system access to prevent any intruder from taking control of IoT devices or disclosing confidential information stored at object or node level. Beltran et al. (2017) introduced SMARTIE, an integrating platform for user-centric secure IoT applications. It preserves user privacy while guaranteeing scalability and efficiency. The proposed platform efficiently provides decentralised access control for IoT devices based on user privacy preferences. The aim of SMARTIE is to facilitate the integration of user-centric privacy and governance within IoT applications in a scalable and efficient mode. The authors highlighted that the proposed application will allow users to control their devices that join the application in terms of sensing and publishing data and enable fine-grained access control rules for their devices whilst deciding who can and cannot be in possession of their device data. The solutions proposed by Burange and Misalkar (2015) and Peters et al. (2019) mitigate privacy risks by providing the final decision maker with the opportunity to finalise network access for the client thereby protecting the privacy of user data. The Peters et al. (2019) study proposed a privacy awareness framework - PrivacyZones, which requires the service provider to share meaningful features of the data collected by their application. The proposed framework was successfully tested using two case study services (Hail-A-Taxi and Get-A-Discount).

Use of AI can improve security and privacy in smart cities. González García et al. (2017) proposed and tested the analysis of pictures through computer vision to detect people in the analysed images. By using different tests, it was found that the system detects pictures with heads and shoulders more accurately in comparison with other images. Additionally, the study found that it is possible to integrate computer vision within IoT networks and that pictures can be used as sensors thereby, helping to improve the security of homes within smart cities. Huerta and Salazar (2019) proposed a framework by using AI and cognitive functions, which is capable of learning to understand, analyse and audit every product in an automated intelligent manner.

Gheisariy et al. (2019) discovered that a number of existing solutions have three major drawbacks. First, applying one static privacy-preserving method for the entire system; second, sending the whole data at once and third, a lack of context-awareness. These aspects can lead to an unacceptable high level of privacy-preserving overhead. In order to deal with these issues, the authors proposed a software-defined networking paradigm that can be directly applied to smart city applications. The Guo et al. (2017) study used an attribute-based trust negotiation scheme for communication between devices within a smart city. The research modelled the trust negotiation process using homographic encryption to guarantee its security. The proposed protocol ensured that a device satisfies its counterparty’s access policy whilst disclosing minimal privacy.

The cloud-oriented architecture solution proposed in Krichen and Alroobaea (2019) posited a new model-based framework for testing security properties of IoT based systems within smart cities by describing the strategy adopted by the malicious party which intends to violate the security of the considered IoT system. The Han et al. (2019) study developed a lightweight and privacy-preserving public cloud-auditing scheme for smart cities that does not require bilinear pairings. The proposed pairing-free scheme allowed a third-party auditor to generate authentication meta-data on behalf of users and provided data privacy against third-party auditors and cloud service providers. The Han et al. study found that the proposed scheme is more secure and efficient in comparison with the existing public cloud auditing schemes.

Aspects of the literature have focused on security and privacy systems for the business environment. The Avgerou et al. (2016) study proposed the deployment of a Privacy-ABCs based authentication system into a generic eBusiness model that provides collective intelligence based eServices within Smart Cities. The model entailed the collective intelligence-interactions between citizens and facilities of smart cities and a privacy-enhancing technology titled attribute-based credentials. By using this approach buying history and consumer behaviour of citizens remains private while interacting with the eCommerce based ecosystem. The research outlined in Cagliero et al. (2015) presented a non-emergency data analyser study the perception of citizens on urban security in the context of the business environment.

The role of software within smart cities is essential, but it brings some privacy and security issues such as exchange of application data, problems related to tracking, effects of hacking, authentication of datasets, increase in personal data thefts, access to information in data centres, effect of other applications and economic pressure (Sen et al. 2013). The study by Sucasas et al. (2018) proposed an OAuth 2.0 based protocol for smart city mobile applications that addressed the user privacy issue by integrating a pseudonym-based signature scheme and a signature delegation scheme into the OAuth 2.0 protocol flow. The proposed solution allows users to self-generate user-specific and app-specific pseudonyms on-demand and ensures privacy-enhanced user authentication at the Service Provider side.

Some studies criticised the existing work and proposed new solutions (Gope et al. 2018; Xie and Hwang 2019; Zang et al. 2017). For example, Xie and Hwang (2019) showed that the scheme proposed by Xiao et al. (2017) lacks two-factor security, and suffers from an impersonation attack. To mitigate these problems, an improved roaming authentication protocol with two-factor security was proposed, secured by using an applied pi calculus-based formal validation tool ProVerif demonstrating enhanced efficiency in comparison with some related schemes.

Zang et al. (2017) asserted that the security protocol proposed in Sookhak et al. (2015) has inherent security flaws, thus failing to achieve its original goal. Specifically, this protocol is vulnerable to two types of attacks, namely - replace attack and replay attack. The study showed how a malicious server can deceive data owners to believe that data is being maintained effectively by launching such attacks. Additionally, it described an improved Remote Database Access (RDA) protocol by utilizing algebraic signatures to fix security flaws. The solution employed the rank-based Merkle Hash Tree to achieve verifiable dynamic data operations. Moreover, the study provided detailed security proof of the proposed RDA protocol. Gope et al. (2018) criticised existing Radio-Frequency IDentification (RFID) technology for its compromise on privacy and forgery detection problems and heavy computation burden due to the very limited computation capability of RFID tags. The study attempted to address these issues by proposing an RFID-based authentication architecture for distributed IoT applications suitable for smart environments.

### Operational Vulnerabilities for Smart Cities

Data within smart city applications should be able to withstand modification, disruption, inspection, unauthorised access, disclosure and annihilation. Basic requirements for security and privacy include confidentiality, integrity, availability, nonrepudiation, access control and privacy (Dewi Rosadi et al. 2018). Smart city residents can face security and privacy issues due to smart city app vulnerabilities, however, without perceived security protection and privacy, the public might hesitate to use smart city mobile applications. Privacy is a core issue within smart cities and one that can be directly linked to the minimal understanding of privacy from local government and business in the way they collect and process personal data. Often they do not provide the community with the opportunity and mechanism for consent (Dewi Rosadi et al. 2018).

Some studies focus on smart city initiatives for specific countries, such as Indonesia (Dewi Rosadi et al. 2018), China (Yang and Xu 2018), and Austria (Dhungana et al. 2015). The research outlined in Dewi Rosadi et al. (2018) explored and analysed the privacy concerns within smart cities in Indonesia, highlighting the complexities of increased amounts of stored and communicated personal information that can be gathered and stored then distributed across multiple devices, services and locations. Yang and Xu (2018) examined applicable laws and regulations in the Chinese context. The authors argued that there is no functional privacy law in China that would apply to most data collected by smart city infrastructure; nor is there any law that would protect any personal data collected under this framework. Some countries (e.g. UK) have recently developed various laws that help legally protect the privacy rights of their citizens (GDPR 2019). For example, the EU General Data Protection Regulation (GDPR) provides essential guidance to achieve a fair balance between the interests of IoT providers and users. Wachter (2018) argues that GDPR standards need further specification and implementation into the design of IoT technologies.

Other legal issues such as jurisdiction, governance of data and handling consent in smart cities were highlighted by Grieman (2019). Dhungana et al. (2015) discussed cases from the Vienna smart city project outlining a number of data analytics scenarios to describe the measures adopted for secure handling of data. The study identified the following privacy and security challenges: privacy guarantees, flexibility privacy policies, anonymity, and data provenance. The project used anonymization, data aggregation, data perturbation and randomization, and cryptographic framework for data mining. It was found that the chosen solution had an impact on the public awareness and acceptability of the smart city project.

While Aldairi and Tawalbeh (2017) and Ferraz and Ferraz (2014b) focused on infrastructure security issues such as eavesdropping, theft, denial of service, information tracking, user/citizens data losses and other threats (e.g. hardware failure, software crash, environment and nature behaviour), Baig et al. (2017) presented a holistic view of the security landscape of a smart city by identifying security threats. The study argued that different components of smart cities have a number of security threats. For example, smart grids have protocol vulnerabilities, privacy, eavesdropping, and attacks on internet-connected devices. Building Automation systems have security threats such as highly trusted devices, long device lifecycle lack of source authentication, and insecure protocols. For unmanned aerial vehicles, security threats include communication interaction, communication injection, and communication jamming. For smart vehicles issues could be related to a physical threat, communication interception, data security, and DoS. For IoT sensors, security threats could include maintaining the confidentiality of data, secure communication, data management, data storage, sensor failure and remote exploitation. Finally, for cloud platform security - threats could include data leakage, malicious insider threats, insecure API, DoS, malware injection attacks, system and application vulnerabilities.

Studies have analysed many of the security threats within smart cities offering a number of potential solutions. Kitchin and Dodge (2019) suggested a wider set of systemic interventions such as security-by-design, remedial security patching and replacement, the formation of core security and computer emergency response teams, a change in procurement procedures, and continuing professional development. Srivastava et al. (2017) presented some smart solutions to safety and security which are enhanced by the use of Artificial Intelligence (AI). The solutions which are already in place in some developed smart cities are gunshot detection sensors, video surveillance and analytics, drones, and cybersecurity. However, Vattapparamban et al. (2016) argue that the use of these technologies (e.g. drones) can result in a number of technical and societal concerns regarding cybersecurity, privacy, and public safety.

While most of the studies in this area focus on privacy and security risks, Velasquez et al. (2018) argued that it is important to consider natural risks when planning smart cities. The study proposed a new architecture which includes the fundamental services that need to be preserved and prioritised within a smart city. The research undertaken by Techatassanasoontorn and Suo (2010) utilising archival and interview data found five risk categories in the municipal broadband project. The interviews were conducted with public policy experts, telecom consultants, and government officers. The following risks were identified: (1) social-political; (2) approval; (3) financial; (4) technical; (5) partnership. The study identified that some of the categories of threats such as socio-political risks have an impact on each other and argue that risk management and risk mitigation strategies are required to take a more holistic view of all threats and their interconnections instead of focusing on each type of risk separately.

### Use and Adoption of Smart Services by Citizens (Success of Smart Services

A number of studies highlighted the importance of perceived security and privacy in smart cities services by citizens (Belanche-Gracia et al. 2015; Chatterjee et al. 2018; Cilliers and Flowerday 2014; Cilliers and Flowerday 2015; Van Heek et al. 2016; van Zoonen 2016). It was found that perceived security and privacy significantly affect the use and adoption of smart services by citizens. For example, Belanche-Gracia et al. (2015) investigated attitudes towards continuance relating to smartcards, user identification, access to local facilities, and payment of small fees for basic services. By using data collected from 398 individuals living in Spain and using Partial Least Square (PLS) analysis, it was found that security has a significant effect on continuance intention of smart card use. Surprisingly, it was found that privacy does not influence intention. It can be explained that the personal information appearing in the card is very limited. As a result, cardholders did not seem to be perturbed by the privacy issues related to smartcard use. By taking into account the fact that security has a positive effect on the use of smart card services, it is advised that public managers and smart card developers need to guarantee smartcard security in order to make the service useful and worthy of the use for citizens.

Some studies claim that the success of the crowdfunding project depends on the perceived trustworthiness of the crowdsourcing system (Cilliers and Flowerday 2015; Cilliers and Flowerday 2014). Cilliers and Flowerday (2015) examined the relationships between the privacy, information security and perceived trustworthiness of crowdsourcing system in a smart city. By using a survey of 361 participants from South Africa the study found a positive relationship between information security and the perceived trustworthiness of a crowdsourcing system. Thus, the privacy concerns of citizens using a crowdsourcing process can be addressed by increasing the perceived trustworthiness and the information security of the system. Another study by Cilliers and Flowerday (2014) investigated factors which mitigate information security concerns of citizens participating in a public safety-participatory crowdsourcing smart city project. Via the analysis of data from completed questionnaires, the study found that security aspects of the system such as confidentiality, integrity and availability, were raising the concerns of citizens that took part in the crowdsourcing project. These findings highlight the importance of implementing legislation and adequate technology to protect the confidentiality of citizens. Additionally, it is important to educate citizens about the relevant information security controls to help protect information integrity.

Studies differ on the extent of privacy concerns depending on the type of technologies, data usage and location. According to van Zoonen (2016) there are four areas of concern amongst people in smart cities that range from low levels (impersonal data, service purpose), to extremely high (personal data, surveillance purpose). The study explored how specific technologies (smart bin, smart parking), and data usage (predictive policing, social media monitoring) may produce various privacy concerns. Van Heek et al. (2016) focused on the location where the technology is used. By using survey data from 119 users the study found that surveillance technologies are accepted in the location where crime threat is present such as public spaces (e.g. train stations or parks); whereas, attitudes were different in relation to more private spaces as the perceived threat is deemed to be relatively low and the use of cameras or microphones is distinctly rejected.

While some of the studies just looked citizens use and adoption of smart services, Chatterjee et al. (2018) focused also on IT staff. The study argued that for successful implementation of smart cities it is important to consider the level of expertise of the internal IT staff to develop and support the smart services and citizens’ participation to use these smart services with full confidence and be less worried about security and privacy issues. By using 230 respondents living in India and PLS for data analysis it was found that experience and knowledge of IT authority significantly affect system security and privacy policy which internally affects operational efficiency and user experience which finally has an impact on adoption of IT services in smart cities. Thus, it is important to have proper training and readiness for both categories. Citizens should have proper awareness and understanding of the system while IT authority should have good training and communicate effectively with citizens.

### Use of Blockchain in Smart Cities

Studies have proposed the use of blockchain technologies to solve some of the issues of privacy and security in smart cities (Mora et al. 2019; Noh and Kwon 2019; Ramos and Silva 2019). Mora et al. (2019) proposed that blockchain-based solutions should be implemented within smart cities to help reduce levels of privacy exposure while providing the user benefits such as trusted transactions and better data control. The study conducted experiments to measure the privacy exposure and quantify the number of cloud resources if IoT technology is implemented within blockchain smart contracts to validate the identity, operation and privacy of citizens. The authors argue that the adoption curve to implement blockchain in large-scale scenarios will take time due to obstacles relating to laws and societal norms. There are a number of factors that can affect the use of blockchain technologies in smart cities such as user factors, technical system factors, and legal as well as institutional factors (Noh and Kwon 2019). Ramos and Silva (2019) state that it is important to understand government processes within a political and legal framework while implementing blockchain technologies. The study argues that blockchain might not always be the best solution for data processing and that it is important mitigate some of the risks for data subjects when processing is carried out via blockchain.

### Social Media and Smart Cities

Data collected from online social networks (e.g. Facebook. Instagram, LinkedIn) provides social, economic, and cultural information which can be used by government, policymakers, authorities, and commercial industries. It can help them better understand market trends and behavioural patterns which influence the individual dynamics through open data sources. However, online social networks can cause threats to privacy issues. Moustaka et al. (2019) aimed to address these issues by revealing the risks, threats and individual behaviour associated with online social networks to improve privacy, security and increase community engagement in smart cities. The study identified that the main vulnerabilities of online social networks use are the risks which threaten an individual’s identity, anonymity, personal space, privacy and communication, and security threats caused by third parties. The study states that smart city stakeholders aim to enhance protection of individuals during social networking and encourage the participation in social networks to design and implement appropriate policies which take into account stages of individual behaviour in social networks. The study proposed a relationship model tested and validated by an empirical study in the smart city of Trikala in Greece.

## Discussion - Smart City Challenges

The advancement of smart cities throughout the world has enabled citizens to communicate with multi-levels of government, gain access to services and enhance the efficiency and effectiveness of system interaction leading to improvements in economic prosperity and quality of life (Nam and Pardo 2011; Yeh 2017). However, significant challenges remain in areas relating to privacy, security and risk from a number of stakeholder perspectives. The literature highlights how these many challenges can impact the benefits to citizens and expose vulnerabilities that could be exploited by third party organisations (Ahmed et al. 2016; Baig et al. 2017). Despite the significant opportunities and merits of IoT enabled smart environments, security and privacy are key factors that expose real threats to the secure operation of smart cities infrastructure (Ahmed et al. 2016).

The evolution of technology and transition towards an integrated digital society is likely to impact many cultural and societal aspects of daily life where the challenges of maintaining human interaction and a sense of belonging and identity, are an integral aspect of being human (Monzon 2015). The literature has presented how these factors can potentially constrain further development and jeopardise the realisation of benefits to citizens and wider stakeholders; where perhaps the interaction and human related factors are omitted (Chauhan et al. 2016; Degbelo et al. 2016). These challenges in the context of the security, privacy and risk within smart cities are outlined below:

### Trust Challenges

The multidimensional nature of smart city initiatives necessitates the need for interaction with human and social capital using technological solutions as the tool to achieve smart city goals in improving the quality of life for its citizens (Monzon 2015). The concept of trust is critical within the smart city context, as the integrated design and underlying technical architecture, is heavily reliant on the efficient and secure communication of large amounts of data. New applications and services have emerged that use this data as they facilitate the communication between citizens and different levels of government. The power of GPS based tracking data, integrated with detailed personal information on shopping habits, location, personal interests, communicated via IoT based infrastructure, poses significant security and privacy concerns (Abosaq 2019; Elmaghraby and Losavio 2014). The new modes of urban governance that exist within the smart city infrastructure, expose a number of new threats to user and network data privacy and confidentiality, in the context of communication and establishing trust between devices (Cho 2012).

The tensions between the development of systems that seek to develop more effective modes of governance and efficiencies, with the potential impact on privacy and confidentiality concerns, is likely to increase. The large amount of data processed by corporate systems within smart city networks, means that organisations and governance authorities will need to balance the benefits of data analytics with individual and societal rights to maintain trust in government (Kitchin 2014). Long lasting trust is reliant on making citizens the central focus in exploiting IoT and smart city opportunities whilst recognizing that firms need to operate within the whole city ecosystem and its many heterogeneous stakeholders (Scuotto et al. 2016).

The development of smart cities can potentially reinforce existing social inequalities and societal bias rather than breaking down these barriers to greater inclusion and integration (Datta 2015). Although the smart city of the near future should be able to achieve significant benefits by seamlessly connecting between both the material and digital world (Nam and Pardo 2011), there exists the risk that this may also effectively disenfranchise sectors of the population that either cannot, or do not wish to interact with the smart city digital infrastructure. This digital disenfranchisement is likely to impact specific demographics who may have concerns over privacy, security or a reticence to engage with new processes and systems, citing security or personal data risks (Joss 2018). There is a risk that smart city initiatives perhaps serve the needs of the technologically astute, the wealthy and effectively institute control and regulation on citizens, particularly those in the periphery of society and social class, less educated in many of the safety and security complexities (Gil-Garcia 2012; Kitchin 2016).

### Operational and Transition Challenges

Few cities throughout the world are designed and developed from the ground up to be smart. The reality is that for many cities throughout the world the key challenge is to develop a managed and strategic transition to smart capability whilst minimising disruption for existing stakeholders and mitigating threats to system integrity and data security. The smart city initiatives within Hong Kong illustrate these complexities where the rapid changes to infrastructure to develop smart buildings are effectively constructed over local ecosystems and are not designed to integrate with the wider bio-region to benefit the whole city (Cugurullo 2018). The implications for the rapid pace of development of smart cities throughout the world indicates the crucial role for case study based research to develop a reflection based narrative (Kitchin 2015). The Brussels case study within Walravens (2015), analyses the mobile technological challenges in the context of platform complexities, highlighting the criticality of including a user perspective when developing new tools and applications. The transitional complexities experienced by the city of Ghent in Belgium where the move to smart based processes was deemed to be problematic, indicates the importance of ambition and leadership driven change (Van den Bergh and Viaene 2015). The issues relating to security and privacy can all too often be side-lined where poor governance has led to inadequate risk assessment of the threats to the operation of the smart city.

The underlying threat of Smart technologies should be viewed from a social and political perspective that reflects the aims and biases of system designers. The one size fits all technologically focussed approach generally applied to smart city initiatives, fails to recognise that solutions are contingent on a number of human centred factors such as: cultural context, history and sense of place (Kitchin 2015). These factors could effectively be “designed in” for new blank canvas smart city developments such as Masdar in Abu Dhabi. However, the adaptation of existing city infrastructure for smart initiatives is problematic leading to increased threats to the security, privacy and sustainability of operations (Awad et al. 2019; Cugurullo 2018). The increasing complexity of interconnected systems poses significant challenges to designing and developing smart city capability that offers robust operational infrastructure (Kitchin 2014). The reality of retrofitting smart cities initiatives effectively integrating the concepts of cities and digital systems, each distinctly highly complex with contingent systems, then binding them together to create environments inherently prone to security vulnerabilities, is a significant challenge and risk to operational capability (Kitchin and Dodge 2011; Townsend 2013).

### Technological Challenges

The significant developments within wireless and sensor-based technologies has paved the way for the widespread deployment of IoT based technologies within smart city environments (Ahmed et al. 2016). The operation of the smart city requires the integration of key technologies such as: IoT, big data, sensors, machine learning and GPS based applications, all of which raise significant threats to the security and integrity of citizen related data. Systems are required to be technologically rigorous with adequate security mechanisms to prevent data breaches and expose vulnerabilities. The significant risks and inherent complexities of data acquisition, storage and transmission from smart city infrastructure such as: smart grids, building automation systems, Unmanned Aerial Vehicles (UAV) and Electric Vehicles (EVs), remain largely unaddressed (Baig et al. 2017). Smart city network architectures are likely to need to cater for the ever-increasing volumes of data from a heterogeneous set of interaction devices, sensors and systems (Silva et al. 2018). The low quality and somewhat disparate nature of smart city data can be detrimental to the effectiveness and accuracy of critical systems. These factors pose additional risk in the context of large-scale deployment of multi-vendor systems and devices with state of the art technologies (Barnaghi et al. 2015; Nam and Pardo 2011).

The limited number of case studies that have explored some of the key factors relating to risks associated with privacy and security within smart cities, highlights the threats from poorly defined roles and responsibilities of different parties, lack of common understanding of key security requirements not shared between parties, flexibility of privacy policies, anonymity, and data provenance as key factors (Dhungana et al. 2015; Vitunskaite et al. 2019). The movement of citizens around the smart city will trigger sensors and network devices that will communicate data about their location, habits and activities as users interact with mobile applications and interact with the smart city infrastructure. However, catering for privacy aspects within the smart city context seems to be a significant technological challenge to designers and system builders. Systems that protect privacy should be closely aligned with continuous security requirements where implementation is essential for trust and wellbeing within smart cities (Elmaghraby and Losavio 2014).

The smart city discourse has tended to distil the concept, generally to a set of issues where cities can use technology to ensure crime is reduced, traffic is more efficient and environmentally friendly, reduced energy consumption, and people lead more healthy and fulfilled lives. However, as highlighted in Kitchin (2016), the technology seems to be the starting point rather than a mechanism to address problems and deliver benefits to city stakeholders. The use of technology is viewed as being of central importance to smart cities but the integration of ICT into urban infrastructure alone - does not make the city smart if the human and social capital as well as wider economic policy and management of urban development are not factored in (Caragliu et al. 2009; Hollands 2008; Kitchin 2014). The realities of smart city initiatives that effectively create innovation ecosystems enabling citizens and communities to interact with public authorities and knowledge developers, highlights that “people rather than technology are the true actors of urban “smartness” (Oliveira and Campolargo 2015). The debate around the underlying human centred factors indicates that in the context of security and privacy within the smart city, the critical emphasis should be one that assesses the benefits and risks from the people perspective when deciding outcomes.

### Sustainability Challenges

Although studies have criticised smart and eco focused city initiatives for the fragmented nature of their sustainable urban development in Hong Kong and Masdar City within Abu Dhabi (Cugurullo 2018), the smart city can offer the potential to integrate low carbon transport infrastructure to deliver the flexible mobility needs of citizens. The digitization of many aspects of smart city management offers significant opportunities within an IoT-enabled waste management infrastructure for the efficient collection, transportation and recycling of materials (Anagnostopoulos et al. 2017). Smart environments are likely to deliver significant innovation and respond to the challenges of greater urbanization with an increased focus on sustainability, energy distribution, mobility, health and security (Klein and Kaefer 2008). The increasing use of technology to advance sustainability and manage natural resources, has the potential to make a significant impact on the quality of life for citizens, whilst aligning with the ethical demands of modern society (Chourabi et al. 2012; Höjer and Wangel 2015).

One of the challenges for sustainability initiatives within smart cities is the need to take account of the human behaviours and motivation of citizens. The Singapore case study analysed in Bhati et al. (2017), highlights the criticality of citizens feelings of safety and security, asserting that these factors must be formally managed before citizens can focus on sustainability initiatives. Citizen quality of life and sense of community are interrelated with sustainability and economic growth, where wise management of economic resources, focus on smart mobility and analysis of how people actually live, is key for a sustained change in behaviours (Bifulco et al. 2016; Lee et al. 2014). The concept of the smart community is based on the convergence of citizens with smart homes and buildings, water and waste management systems to maximise the benefits of the smart city in the optimisation of energy consumption, and reduction of carbon emissions (Silva et al. 2018).

The management of many aspects of sustainability initiatives within smart cities via complex IoT focussed technology, exposes organisations to the threats from failure due to natural disasters but also through network unavailability and breaches of security. Designing for smart city sustainability includes rapid recovery strategies to overcome failure and to revert the city operations back to normal with minimal cost and impact on operational efficiency (Silva et al. 2018).

### Smart City Interaction Framework

**Fig. 3:**
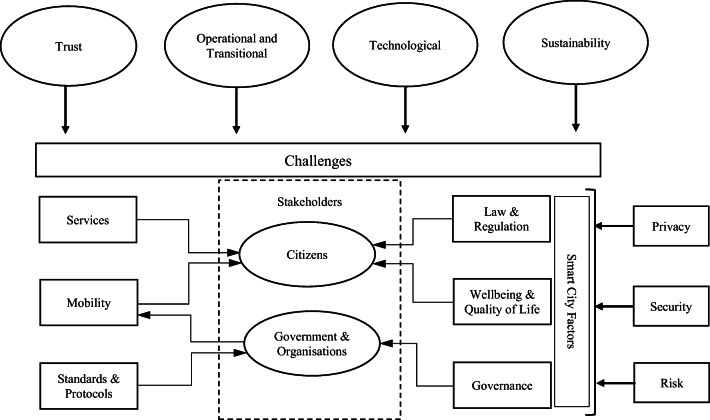
Smart cities security & privacy framework

The framework presented in Fig. 3 highlights the numerous interdependencies between the many identified factors and challenges within smart cities and integrates these into a single model.

The framework details the impact of the key challenges on the various operational functions within the smart city and contextualises the interaction with key factors such as services and mobility from the stakeholder perspective. The complexities inherent within privacy, security and risk within smart cities are represented across all aspects of the model. These aspects are integral to smart city operations requiring effective processes and procedures at all levels of transactions and interactions with the smart city infrastructure.

The key challenges for smart cities namely trust, operational and transitional, technological and sustainability are outlined in the framework, signifying the pressure on smart city designers and integrators to retain focus on these elements. The key challenge of engendering trust from citizens is integral to expanding operational reach and effective interaction from smart city systems and infrastructure. Without trust from all stakeholder levels, citizens will be reticent to interact with smart city systems and interfaces. Feelings of digital disenfranchisement and reticence to interact can be manifestations of low levels of trust where citizens have concerns on security or perception of threats relating to personal data integrity (Joss 2018).

The identified smart city factors within the framework namely: services, mobility, standards and protocols, law and regulation, wellbeing, quality of life and governance, are derived from the identified themes and literature review. Each represent the range of factors that need to be in place for the smart city to function effectively. The key stakeholders - citizens, government and organisations, highlights the significant reliance on the human factors and their interaction for the successful operations of smart cities (Kitchin 2015; Scuotto et al. 2016). As cities adopt greater levels of smart capability, the significant challenges as outlined remain. The impact on the lives and wellbeing of citizens is significant, therefore, the success of smart cities and challenge for authorities is the building of trust through privacy and security initiatives. The threats to operational effectiveness of smart cities are numerous and are dependent on many aspects of security policy, education and effectively managing the balance between openness and acceptable levels of intrusion and security. These areas are ongoing challenges for future smart city initiatives.

Based on the discussion above and presented framework (Fig. 3) the following is proposed, which could serve as a basis for future empirical research to validate the proposed framework:

#### Proposition 1

Solving the many technological and sustainability challenges can significantly influence adoption and reduce the risk of operational and citizen interaction issues within smart cities. The inherent risks and complexities associated with smart city infrastructure with its use of smart grids, building automation systems and IoT interaction devices ( Baig et al. 2017; Silva et al. 2018), integrated with large scale, leading edge technology deployment, pose numerous issues for smart city designers and operators (Barnaghi et al. 2015; Nam and Pardo 2011). The success of smart cities is reliant on the interaction from citizens and wider stakeholders (Oliveira and Campolargo 2015), but success is very much dependent on design approaches, operational efficiency, and the human centred approach to assessing benefits. The increasing ethical demands of modern society has greatly impacted how people view smart environments and risks relating to sustainability and wider aspects of society (Chourabi et al. 2012; Höjer and Wangel 2015). Greater adoption of the many changes resulting from smart cities is directly related to a deeper analysis and understanding of how people live and work (Bifulco et al. 2016; Lee et al. 2014), where smart community and smart mobility are integrated with the overall city infrastructure.

#### Proposition 2

Increasing focus on the many issues related to the privacy and security elements of smart city infrastructure, will engender greater levels of trust in smart city operations and smart city system interaction. Factors relating to trust are critical to the success of smart cities and are reliant on the effective management and communication of data. The significant levels of data communicated via IoT based infrastructure, pose significant security and privacy concerns in the minds of citizens (Abosaq 2019; Elmaghraby and Losavio 2014), especially for those demographics that exhibit high levels of reticence to interact with technology (Joss 2018). Designers of smart cities are advised to focus on these factors as they balance the needs of citizen concerns relating to privacy and security with greater access to personal data.

#### Proposition 3

The move from existing traditional infrastructure to one encompassing smart city initiatives, poses significant risk to designers and planners unless sufficient focus is maintained on the human related transition factors. The reality that most smart city projects are effectively a retro-fit of smart initiatives within an existing infrastructure, highlights the criticality of ensuring a key stakeholder perspective when developing new smart systems and levels of interaction. The singular technologically focussed approach generally applied to smart city initiatives, fails to recognise that solutions are contingent on a number of human centred factors (Kitchin 2015), where the adaptation of existing city infrastructure can increase the risk to the security, privacy and sustainability of operations (Awad et al. 2019; Cugurullo 2018). The cultural and societal shift required to transition stakeholders to new smart city operations should not be underestimated.

## Conclusion

This study provides a theoretical review of the smart city literature, focussing on the many threats relating to privacy and security, and how these can impact the operation of smart city processes. A number of emergent themes and significant challenges have been discussed to provide a valuable synthesis of the key factors relating to privacy and security threats within the smart cities literature. We posit this study as a comprehensive and relevant information framework for academics and practitioners analysing the many complexities and issues surrounding smart cities. As a result of the literature review, discussion and developed framework, a number of propositions were developed.

The analysis of the existing literature highlights that generally, studies seem to be lacking quantitative and also qualitative data on the adoption of smart cities. Some studies do include a case element to their research, but the recycling of lessons learned into new smart city initiatives, seems to be a key gap in the literature. The vulnerability of smart city infrastructure to data theft, unauthorised data access, system breaches, virus-based attacks and other threats to operational integrity, are likely to be ongoing as more cities transition to smart capability. The critical factors relating to technical and security governance of smart cities are key factors relating to operational integrity and citizen trust within the smart city infrastructure. Breaches of security that jeopardise data privacy are likely to severely impact citizen engagement and overall trust in smart city systems and services.

The greater focus on sustainability within the smart city infrastructure is likely to impact provision of services, transportation, eating habits and consumerism. The wellbeing and quality of life factors are directly related to these areas as citizens interact with new initiatives and adhere to changes that reduce emissions and reduce our carbon footprint. The links between feelings of safety and security and greater willingness for citizens to transition toward sustainability initiatives, could act as key drivers for smart city authorities to build in these aspects at an early stage.

The ongoing use of technology to provide the infrastructure and interaction that can deliver services to citizens, whilst offering new innovative platforms and systems many of which are available via mobile devices, risks disenfranchising key sectors of the population. The fast pace of technology that can delivery new and exciting ways to interact with healthcare providers, banks insurance companies, utility providers and transport operators, is likely to be a barrier for adoption for older demographics that do not share the same feelings of trust and enthusiasm from younger more technically aware population groups. The challenge for smart city designers and planners as innovations such as blockchain based systems and greater use of AI become integral to system architectures, is to maintain humans in the loop to engender the required levels of trust in security and privacy from citizens.

## Limitations and Future Research Directions

This study is somewhat limited due to the focus on security and privacy that perhaps may exclude a number of the human-centred factors that may impact the further adoption of smart cities. Further research is recommended to better illustrate the “lived in” experience of smart cities from the citizen perspective to quantify the many interaction and day to day operational complexities to engender greater levels of trust from the population. Furthermore, the current study considered only technological solutions to improve security, privacy, and operational threats. However, city infrastructure also includes legal and institutional dimensions. Future research should consider how the legal system can be used to solve trust challenges within smart cities. Additionally, it is advised that future research should focus on solving identified challenges of smart cities (trust challenges namely trust challenges, operational and transition challenges, technological challenges and sustainability challenges), which will greatly benefit further smart city initiatives.

More research is needed on the use of blockchain in smart cities. It is important that new government and industry regulations are developed with help to avoid disputes among the transacting parties (Krawiec et al. 2016). Additionally, it is important to consider the cost of deploying and operating a blockchain-based system within smart cities. Thus, it is important to perform a targeted pilot in order to test the potential cost of a blockchain-based smart city system (Xie et al. 2019), as well as to evaluate holistic models of societal value co-creation that consider thorough cost-benefit analysis to enable sound decision-making by government.

More research is required on smart cities’ complex institutional and technological environments (Gupta et al. 2020). Future studies could explore the role of leadership and orchestration, which manifested through openness, diffusion, and shared vision in smart city ecosystems (Gupta et al. 2020).

The substantial advances in smart city technological infrastructure have provided for many examples of smart city implementations. However, in order to ensure that benefits can be gained at a local level, as well as at a level that transcends the local community to embrace regional, national, and global dimensions. The current issues around heterogeneity, and lack of communication protocol and standardisation, and the mostly proprietary and close nature of such infrastructure, which currently prevent different ‘smart cities’ to communicate, need to be resolved (Allam and Jones 2020). A globally interconnected, transparent network of smart cities, and democratised access to the infrastructure could be a critical tool in tackling global health issues, such as virus outbreak (as in the case of the COVID-19 pandemic) by greatly enabling real-time and globally coordinated urban health management by access to, and monitoring of, a large number of critical sensory outputs. This line of thinking can also be extended to other global issues such as pollution and environmental monitoring, whereby a communicating network of smart cities would provide a step-change in the predictive ability of environmental models, with evident benefits for city dwellers, proactive policy development at regional, national and international levels (with regulatory, legal, economic and wider research implications that will inevitably accompany them), with the design and adoption of countermeasures at scale. The future research could also explore and learn from the successful adoption of information technology enabled citizen-centric services (e.g. Alryalat et al. 2015; Chatterjee and Kar 2018; Chaudrie and Dwivedi 2005; Dwivedi and Williams 2008; Dwivedi et al. 2007, 2017; Kapoor et al. 2014; Rana and Dwivedi 2015; Rana et al. [Bibr CR140][Bibr CR141][Bibr CR147][Bibr CR142][Bibr CR143][Bibr CR144][Bibr CR145][Bibr CR146], 2017; Weerakkody et al. 2007,2009,2017) and other IS success/failure projects (e.g. Hughes et al. [Bibr CR83][Bibr CR82][Bibr CR85][Bibr CR84]) for the proposed smart cities in India.

## References

[CR1] Abi Sen, A. A., Eassa, F. A., & Jambi, K. (2018). Preserving privacy of smart cities based on the fog computing 10.1007/978-3-319-94180-6_18.

[CR2] Abosaq NH (2019). Impact of privacy issues on smart city services in a model smart city. International Journal of Advanced Computer Science and Applications.

[CR3] Ahmed E, Yaqoob I, Gani A, Imran M, Guizani M (2016). Internet-of-things-based smart environments: state of the art, taxonomy, and open research challenges. IEEE Wireless Communications.

[CR4] Ainane, N., Ouzzif, M., & Bouragba, K. (2018). Data security of smart cities. Paper presented at the ACM International Conference Proceeding Series, 10.1145/3286606.3286866.

[CR5] Al-Dhubhani, R., Mehmood, R., Katib, I., & Algarni, A. (2018). Location privacy in smart cities era 10.1007/978-3-319-94180-6_14.

[CR6] Alamaniotis, M., Tsoukalas, L. H., & Buckner, M. (2017). Privacy-driven electricity group demand response in smart cities using particle swarm optimization. Paper presented at the Proceedings – 2016 IEEE 28th International Conference on Tools with Artificial Intelligence, ICTAI 2016, 946–953. 10.1109/ICTAI.2016.0143.

[CR7] Alandjani G (2018). Features and potential security challenges for IoT enabled devices in smart city environment. International Journal of Advanced Computer Science and Applications.

[CR8] Albino V, Berardi U, Dangelico RM (2015). Smart cities: Definitions, dimensions, performance, and initiatives. Journal of urban technology.

[CR9] Aldairi, A., & Tawalbeh, L. (2017). Cyber security attacks on smart cities and associated mobile technologies. Paper presented at the Procedia Computer Science, 109, 1086–1091. 10.1016/j.procs.2017.05.391.

[CR10] Allam, Z., & Jones, D. S. (2020). On the Coronavirus (COVID-19) Outbreak and the Smart City Network: Universal Data Sharing Standards Coupled with Artificial Intelligence (AI) to Benefit Urban Health Monitoring and Management. In *Healthcare* (Vol. 8, No. 1, p. 46). Multidisciplinary Digital Publishing Institute. 10.3390/healthcare8010046 PMC7151018 32120822

[CR11] Alromaihi, S., Elmedany, W., & Balakrishna, C. (2018). Cyber security challenges of deploying IoT in smart cities for healthcare applications. Paper presented at the Proceedings – 2018 IEEE 6th International Conference on Future Internet of Things and Cloud Workshops, W-FiCloud 2018, 140–145. 10.1109/W-FiCloud.2018.00028.

[CR12] Alryalat M, Rana NP, Dwivedi YK (2015). Citizen’s adoption of an e-government system: Validating the extended theory of reasoned action (TRA). International Journal of Electronic Government Research.

[CR13] Alter, S. (2019). Making Sense of Smartness in the Context of Smart Devices and Smart Systems. Information Systems Frontiers, 1–13. 10.1007/s10796-019-09919-9.

[CR14] Anagnostopoulos T, Zaslavsky A, Kolomvatsos K, Medvedev A, Amirian P, Morley J, Hadjieftymiades S (2017). Challenges and opportunities of waste management in IoT-enabled smart cities: a survey. IEEE Transactions on Sustainable Computing.

[CR15] Anthopoulos LG (2015). Understanding the smart city domain: A literature review. Transforming city governments for successful smart cities.

[CR16] Antoine Picon G (2019). Smart cities, privacy and the pulverisation/reconstruction of individuals. European Data Protection Law Review.

[CR17] Antonopoulos, K., Petropoulos, C., Antonopoulos, C. P., & Voros, N. S. (2017). Security data management process and its impact on smart cities’ wireless sensor networks. Paper presented at the South-East Europe Design Automation, Computer Engineering, Computer Networks and Social Media Conference, SEEDA-CECNSM 2017, 10.23919/SEEDA-CECNSM.2017.8088238.

[CR18] Avgerou, A., Nastou, P. E., Nastouli, D., Pardalos, P. M., & Stamatiou, Y. C. (2016). On the deployment of citizens’ privacy preserving collective intelligent ebusiness models in smart cities. *International Journal of Security and its Applications, 10*(2), 171–184. 10.14257/ijsia.2016.10.2.16.

[CR19] Awad, A. I., Furnell, S., Hassan, A. M., & Tryfonas, T. (2019). Special issue on security of IoT-enabled infrastructures in smart cities. Ad Hoc Networks, 92. 10.1016/j.adhoc.2019.02.007.

[CR20] Babdullah, A., Rana, N. P., Ali, A. A., Dwivedi, Y. K., & Lal, B. (2017). Assessing Consumer’s Intention to Adopt Mobile Internet Services in the Kingdom of Saudi Arabia. *AMCIS 2017*, Boston, USA, 10-12th August 2017.

[CR21] Baig ZA, Szewczyk P, Valli C, Rabadia P, Hannay P, Chernyshev M, Peacock M (2017). Future challenges for smart cities: Cyber-security and digital forensics. Digital Investigation.

[CR22] Barnaghi, P. M., Bermudez-Edo, M., & Tönjes, R. (2015). Challenges for Quality of Data in Smart Cities. *J. Data and Information Quality*, *6*(2–3), 6 – 1.

[CR23] Baryshev, G. K., Tutnov, I. A., & Karasevich, A. M. (2016). The prospect and risks of using gas-combined cycle heating in the domestic sector of smart cities. Paper presented at the ACM International Conference Proceeding Series, 22-23-November-2016 4–8. 10.1145/3014087.3014104.

[CR24] Belanche-Gracia D, Casaló-Ariño LV, Pérez-Rueda A (2015). Determinants of multi-service smartcard success for smart cities development: A study based on citizens’ privacy and security perceptions. Government Information Quarterly.

[CR25] Beltran, V., Martinez, J. A., & Skarmeta, A. F. (2017). User-centric access control for efficient security in smart cities. Paper presented at the GIoTS 2017 - Global Internet of Things Summit, Proceedings, 10.1109/GIOTS.2017.8016287.

[CR26] Bernardes, M. B., De Andrade, F. P., & Novais, P. (2018). Smart cities, data and right to privacy: A look from the Portuguese and Brazilian experience. Paper presented at the ACM International Conference Proceeding Series, 328–337. 10.1145/3209415.3209451.

[CR27] Bhati A, Hansen M, Chan CM (2017). Energy conservation through smart homes in a smart city: A lesson for Singapore households. Energy Policy.

[CR28] Bibri SE, Krogstie J (2017). Smart sustainable cities of the future: An extensive interdisciplinary literature review. Sustainable cities and society.

[CR29] Bifulco, F., Tregua, M., Amitrano, C. C., & D.‘Auria, A (2016). ICT and sustainability in smart cities management. *International Journal of Public Sector Management, 29*(2), 132–147.

[CR30] Burange, A. W., & Misalkar, H. D. (2015). Review of internet of things in development of smart cities with data management & privacy. Paper presented at the Conference Proceeding – 2015 International Conference on Advances in Computer Engineering and Applications, ICACEA 2015, 189–195. 10.1109/ICACEA.2015.7164693.

[CR31] Cagliero, L., Cerquitelli, T., Chiusano, S., Garino, P., Nardone, M., Pralio, B., & Venturini, L. (2015). Monitoring the citizens’ perception on urban security in smart city environments. Paper presented at the Proceedings - International Conference on Data Engineering, 2015-June 112–116. 10.1109/ICDEW.2015.7129559.

[CR32] Caragliu, A., Del Bo, C., & Nijkamp, P. (2009). Smart city in Europe. *Journal of Urban Technology*, *18*(2).

[CR33] Chatterjee S, Kar AK (2018). Effects of successful adoption of information technology enabled services in proposed smart cities of India: From user experience perspective. Journal of Science and Technology Policy Management.

[CR34] Chatterjee, S., & Kar, A. K. (2015, August). Smart Cities in developing economies: a literature review and policy insights. In *2015 International Conference on Advances in Computing, Communications and Informatics (ICACCI)* (pp. 2335–2340). IEEE.

[CR35] Chatterjee S, Kar AK, Gupta MP (2017). Critical success factors to establish 5G network in smart cities: Inputs for security and privacy. Journal of Global Information Management.

[CR36] Chatterjee S, Kar AK, Gupta MP (2018). Alignment of IT authority and citizens of proposed smart cities in India: System security and privacy perspective. Global Journal of Flexible Systems Management.

[CR37] Chatterjee S, Kar AK, Dwivedi YK, Kizgin H (2019). Prevention of cybercrimes in smart cities of India: from a citizen’s perspective. Information Technology & People.

[CR38] Chaudrie, J., & Dwivedi, Y. K. (2005). A survey of citizens’ awareness and adoption of e-government initiatives, the ‘Government Gateway’: A United Kingdom perspective. In proceedings of eGovernment Workshop ’05 (eGOV05), September 13 2005, Brunel University, West London, UK. Available at http://citeseerx.ist.psu.edu/viewdoc/download?doi=10.1.1.109.2889&rep=rep1&type=pdf.

[CR39] Chauhan S, Agarwal N, Kar AK (2016). Addressing big data challenges in smart cities: a systematic literature review. info.

[CR40] Cho, Y. I. (2012). Designing Smart Cities: Security Issues. In A. Cortesi, N. Chaki, K. Saeed & S. Wierzchoń (Eds.), *Computer Information Systems and Industrial Management. CISIM 2012* (Vol. 7564). Berlin: Springer. Lecture Notes in Computer Science.

[CR41] Chourabi, H., Nam, T., Walker, S., Gil-Garcia, J. R., Mellouli, S., Nahon, K., … Scholl, H. J. (2012, January). Understanding smart cities: An integrative framework. In 2012 45th Hawaii international conference on system sciences (pp. 2289–2297). IEEE.

[CR42] Cilliers, L., & Flowerday, S. (2014). Information security in a public safety, participatory crowdsourcing smart city project. Paper presented at the 2014 World Congress on Internet Security, WorldCIS 2014, 36–41. 10.1109/WorldCIS.2014.7028163.

[CR43] Cilliers, L., & Flowerday, S. (2015). The relationship between privacy, information security and the trustworthiness of a crowdsourcing system in a smart city. Paper presented at the Proceedings of the 9th International Symposium on Human Aspects of Information Security and Assurance, HAISA 2015, 243–255.

[CR44] Cugurullo F (2018). Exposing smart cities and eco-cities: Frankenstein urbanism and the sustainability challenges of the experimental city. Environment and Planning A: Economy and Space.

[CR45] Datta A (2015). New urban utopias of postcolonial India: ‘Entrepreneurial urbanization’ in Dholera smart city, Gujarat. Dialogues in Human Geography.

[CR46] de Amorim, W. S., Deggau, B., A., do Livramento, Gonçalves, G., da Silva Neiva, S., Prasath, A. R., & Salgueirinho Osório de Andrade Guerra,J.B. (2019). Urban challenges and opportunities to promote sustainable food security through smart cities and the 4th industrial revolution. *Land use Policy*, 87 10.1016/j.landusepol.2019.104065.

[CR47] de Fuentes JM, González-Manzano L, Serna-Olvera J, Veseli F (2017). Assessment of attribute-based credentials for privacy-preserving road traffic services in smart cities. Personal and Ubiquitous Computing.

[CR48] Degbelo A, Granell C, Trilles S, Bhattacharya D, Casteleyn S, Kray C (2016). Opening up smart cities: citizen-centric challenges and opportunities from GIScience. ISPRS International Journal of Geo-Information.

[CR49] Dewi Rosadi, S., Suhardi, S., & Kristyan, S. A. (2018). Privacy challenges in the application of smart city in Indonesia. Paper presented at the 2017 International Conference on Information Technology Systems and Innovation, ICITSI 2017 - Proceedings,, 2018-January 405–409. 10.1109/ICITSI.2017.8267978.

[CR50] Dhungana, D., Engelbrecht, G., Parreira, J. X., Schuster, A., & Valerio, D. (2015). Aspern smart ICT: Data analytics and privacy challenges in a smart city. Paper presented at the IEEE World Forum on Internet of Things, WF-IoT 2015 - Proceedings, 447–452. 10.1109/WF-IoT.2015.7389096.

[CR51] Dwivedi YK, Williams MD (2008). Demographic influence on UK citizens’ e-government adoption. Electronic Government, an International Journal.

[CR52] Dwivedi, Y.K., Rana, N.P., Jeyaraj, A., Clement, M., & Williams, M.D. (2017). Re-examining the Unified Theory of Acceptance and Use of Technology (UTAUT): Towards a Revised Theoretical Model. *Information Systems Frontiers, 21*(3), 719–734.

[CR53] Dwivedi YK, Hughes L, Ismagilova E, Aarts G, Coombs C, Crick T, Galanos V (2019). Artificial Intelligence (AI): Multidisciplinary perspectives on emerging challenges, opportunities, and agenda for research, practice and policy. International Journal of Information Management.

[CR54] Dwivedi YK, Khan N, Papazafeiropoulou A (2007). Consumer adoption and usage of broadband in Bangladesh. Electronic Government, an International Journal.

[CR55] Efthymiopoulos, M. (2015). Cyber-security in smart cities: The case of Dubai. Journal of Innovation and Entrepreneurship, 5(1) 10.1186/s13731-016-0036-x.

[CR56] Elmaghraby AS, Losavio MM (2014). Cyber security challenges in smart cities: Safety, security and privacy. Journal of Advanced Research.

[CR57] Evans, L. (2018). The privacy parenthesis: Private and public spheres, smart cities and big data. Creating smart cities (pp. 194–204) 10.4324/9781351182409.

[CR58] Ferdowsi, A., Saad, W., Maham, B., & Mandayam, N. B. (2017). A colonel blotto game for interdependence-aware cyber-physical systems security in smart cities. Paper presented at the Proceedings – 2017 2nd International Workshop on Science of Smart City Operations and Platforms Engineering, in Partnership with Global City Teams Challenge, SCOPE 2017, 7–12. 10.1145/3063386.3063765.

[CR59] Ferraz, F. S., & Ferraz, C. A. G. (2014a). More than meets the eye in smart city information security: Exploring security issues far beyond privacy concerns. Paper presented at the Proceedings – 2014 IEEE International Conference on Ubiquitous Intelligence and Computing, 2014 IEEE International Conference on Autonomic and Trusted Computing, 2014 IEEE International Conference on Scalable Computing and Communications and Associated Symposia/Workshops, UIC-ATC-ScalCom 2014, 677–685. 10.1109/UIC-ATC-ScalCom.2014.143.

[CR60] Ferraz, F. S., & Ferraz, C. A. G. (2014b). Smart city security issues: Depicting information security issues in the role of an urban environment. Paper presented at the Proceedings – 2014 IEEE/ACM 7th International Conference on Utility and Cloud Computing, UCC 2014, 842–847. 10.1109/UCC.2014.137.

[CR61] Fido Alliance. (2019). FIDO Authentication, Accessed on 15.11.2019https://fidoalliance.org/fido-authentication/.

[CR62] Forbes (2019). These are the smartest cities in the world for 2019. Accessed on 12.1.2019, https://www.forbes.com/sites/iese/2019/05/21/these-are-the-smartest-cities-in-the-world-for-2019/.

[CR63] Galdon-Clavell G (2013). Not so) smart cities?: The drivers, impact and risks of surveillance enabled smart environments. Science and Public Policy.

[CR64] GDPR (2019). Data protection rules as a trust-enabler in the EU and beyond – taking stock. (COM/2019/374). Available at: https://ec.europa.eu/info/law/law-topic/data-protection_en. Accessed on 27 March 2020.

[CR65] Gheisariy M, Wang G, Khanz WZ, Fernández-Campusano C (2019). A context-aware privacy-preserving method for IoT-based smart city using software defined networking. Computers and Security.

[CR66] Gil-Garcia, J. R. (2012). Towards a smart State? Inter-agency collaboration, information integration, and beyond. *Information Polity, 17*(3), 4), 269–280.

[CR67] González García, C., Meana-Llorián, D., Pelayo, G.-Bustelo, B. C., Lovelle, C., J. M., & Garcia-Fernandez, N. (2017). Midgar: Detection of people through computer vision in the internet of things scenarios to improve the security in smart cities, smart towns, and smart homes. *Future Generation Computer Systems, 76*, 301–313. 10.1016/j.future.2016.12.033.

[CR68] Gope, P., Amin, R., Islam, H., Kumar, S. K., N., & Bhalla, V. K. (2018). Lightweight and privacy-preserving RFID authentication scheme for distributed IoT infrastructure with secure localization services for smart city environment. *Future Generation Computer Systems, 83*, 629–637. 10.1016/j.future.2017.06.023.

[CR69] Grieman, K. (2019). Pedestrian curiosity: A brief examination of consent and privacy in swath section smart city spaces. Paper presented at the CEUR Workshop Proceedings, 2323.

[CR70] Guo J, Ma J, Li X, Zhang J, Zhang T (2017). An attribute-based trust negotiation protocol for D2D communication in smart city balancing trust and privacy. Journal of Information Science and Engineering.

[CR71] Gupta A, Panagiotopoulos P, Bowen F (2020). An orchestration approach to smart city data ecosystems. Technological Forecasting and Social Change.

[CR72] Gupta P, Chauhan S, Jaiswal MP (2019). Classification of smart city research-a descriptive literature review and future research agenda. Information Systems Frontiers.

[CR73] Gupta S, Drave VA, Bag S, Luo Z (2019). Leveraging smart supply chain and information system agility for supply chain flexibility. Information Systems Frontiers.

[CR74] Habibzadeh H, Soyata T, Kantarci B, Boukerche A, Kaptan C (2018). Sensing, communication and security planes: A new challenge for a smart city system design. Computer Networks.

[CR75] Han J, Li Y, Chen W (2019). A lightweight and privacy-preserving public cloud auditing scheme without bilinear pairings in smart cities. Computer Standards and Interfaces.

[CR76] Heidt M, Gerlach JP, Buxmann P (2019). Investigating the security divide between SME and large companies: How SME characteristics influence organizational IT security investments. Information Systems Frontiers.

[CR77] Hiller JS, Blanke JM (2017). Smart cities, big data, and the resilience of privacy. Hastings Law Journal.

[CR78] Höjer M, Wangel J (2015). Smart sustainable cities: definition and challenges. ICT innovations for sustainability.

[CR79] Hollands RG (2008). Will the real smart city please stand up? Intelligent, progressive or entrepreneurial?. City.

[CR80] Huang, Q., Wang, L., & Yang, Y. (2017). Secure and privacy-preserving data sharing and collaboration in mobile healthcare social networks of smart cities. *Security and Communication Networks*, 2017 10.1155/2017/6426495.

[CR81] Huerta, J., & Salazar, P. (2019). Audit process framework for data protection and privacy compliance using artificial intelligence and cognitive services in smart cities. Paper presented at the 2018 IEEE International Smart Cities Conference, ISC2 2018, 10.1109/ISC2.2018.8656877.

[CR82] Hughes DL, Dwivedi YK, Rana NP (2017). Mapping IS failure factors on PRINCE2® stages: An application of interpretive ranking process (IRP). Production Planning & Control.

[CR83] Hughes DL, Dwivedi YK, Rana NP, Simintiras AC (2016). Information systems project failure–analysis of causal links using interpretive structural modelling. Production Planning & Control.

[CR84] Hughes DL, Rana NP, Dwivedi YK (2020). Elucidation of IS project success factors: an interpretive structural modelling approach. Annals of Operations Research.

[CR85] Hughes DL, Rana NP, Simintiras AC (2017). The changing landscape of IS project failure: An examination of the key factors. Journal of Enterprise Information Management.

[CR86] Hughes L, Dwivedi YK, Misra SK, Rana NP, Raghavan V, Akella V (2019). Blockchain research, practice and policy: Applications, benefits, limitations, emerging research themes and research agenda. International Journal of Information Management.

[CR87] Ismagiloiva, E., Hughes, L., Rana, N., & Dwivedi, Y. (2019a). Role of Smart Cities in Creating Sustainable Cities and Communities: A Systematic Literature Review. In International Working Conference on Transfer and Diffusion of IT (pp. 311–324). Springer, Cham.

[CR88] Ismagilova E, Hughes L, Dwivedi YK, Raman KR (2019). Smart cities: Advances in research—An information systems perspective. International Journal of Information Management.

[CR89] Jameel, T., Ali, R., & Ali, S. (2019). Security in modern smart cities: An information technology perspective. Paper presented at the 2019 2nd International Conference on Communication, Computing and Digital Systems, C-CODE 2019, 293–298. 10.1109/C-CODE.2019.8681021.

[CR90] Janssen MFWHA, Luthra S, Mangla S, Rana NP, Dwivedi YK (2019). Challenges for adopting and implementing IoT in smart cities: An integrated MICMAC-ISM approach. Internet Research.

[CR91] Joss S (2018). Future cities: asserting public governance. Palgrave Communications.

[CR92] Kapoor KK, Dwivedi YK, Williams MD (2014). Innovation adoption attributes: a review and synthesis of research findings. European Journal of Innovation Management.

[CR93] Kar AK, Ilavarasan V, Gupta MP, Janssen M, Kothari R (2019). Moving beyond Smart Cities: Digital Nations for Social Innovation & Sustainability. Information Systems Frontiers.

[CR94] Karasevich, A. M., Tutnov, I. A., & Baryshev, G. K. (2016). The prospects of application of information technologies and the principles of intelligent automated systems to manage the security status of objects of energy supply of smart cities. Paper presented at the ACM International Conference Proceeding Series, 22-23-November-2016 9–14. 10.1145/3014087.3014111.

[CR95] Khan, Z., Pervez, Z., & Ghafoor, A. (2014). Towards cloud based smart cities data security and privacy management. Paper presented at the Proceedings – 2014 IEEE/ACM 7th International Conference on Utility and Cloud Computing, UCC 2014, 806–811. 10.1109/UCC.2014.131.

[CR96] Khedr AM, Osamy W, Salim A, Salem A (2019). Privacy preserving data mining approach for IoT based WSN in smart city. International Journal of Advanced Computer Science and Applications.

[CR97] Kitchenham, B. A. (2004). Procedures for Performing Systematic Reviews. Joint technical report, computer science department, Keele University (TR/SE0401) and National ICT Australia Ltd. (0400011T.1). Available at: http://www.inf.ufsc.br/~aldo.vw/kitchenham.pdf. Accessed on 27 February 2020.

[CR98] Kitchin R (2014). The real-time city? Big data and smart urbanism. Geo Journal.

[CR99] Kitchin R (2015). Making sense of smart cities: addressing present shortcomings. Cambridge Journal of Regions, Economy and Society.

[CR100] Kitchin, R. (2016). Reframing, reimagining and remaking smart cities. Routledge.

[CR101] Kitchin R, Dodge M (2011). Code/Space: Software and Everyday Life.

[CR102] Kitchin R, Dodge M (2019). The (in)security of smart cities: Vulnerabilities, risks, mitigation, and prevention. Journal of Urban Technology.

[CR103] Klein, C., & Kaefer, G. (2008, September). From smart homes to smart cities: Opportunities and challenges from an industrial perspective. In *International Conference on Next Generation Wired/Wireless Networking* (pp. 260–260). Springer, Berlin, Heidelberg.

[CR104] Komninos, N. (2013). “What makes cities intelligent?“. In Deakin, Mark (ed.). Smart Cities: Governing, Modelling and Analysing the Transition. Taylor and Francis. p. 77. ISBN 978-1135124144.

[CR105] Krawiec, R. J., Barr, D., Killmeyer, J., Filipova, M., Nesbitt, A., Israel, A., Quarre, F., Fedosova, K., & Tsai, L. (2016) “Blockchain: Opportunities for health care,” *CP Transaction. Available at*: https://www.colleaga.org/sites/default/files/4-37-hhs_blockchain_challenge_deloitte_consulting_llp.pdf. Accessed on 28 March 2020.

[CR106] Krichen, M., & Alroobaea, R. (2019). A new model-based framework for testing security of IoT systems in smart cities using attack trees and price timed automata. Paper presented at the ENASE 2019 - Proceedings of the 14th International Conference on Evaluation of Novel Approaches to Software Engineering, 570–577.

[CR107] Krichen, M., Cheikhrouhou, O., Lahami, M., Alroobaea, R., & Jmal Maâlej, A. (2018). Towards a model-based testing framework for the security of internet of things for smart city applications 10.1007/978-3-319-94180-6_34.

[CR108] Lai J, Mu Y, Guo F, Susilo W, Chen R (2017). Fully privacy-preserving and revocable ID-based broadcast encryption for data access control in smart city. Personal and Ubiquitous Computing.

[CR109] Lee JH, Hancock MG, Hu MC (2014). Towards an effective framework for building smart cities: Lessons from Seoul and San Francisco. Technological Forecasting and Social Change.

[CR110] Lepinski, M., Levin, D., McCarthy, D., Watro, R., Lack, M., Hallenbeck, D., & Slater, D. (2016). Privacy-enhanced android for smart cities applications 10.1007/978-3-319-33681-7_6.

[CR111] Li H, Zhu H, Choi BJD (2015). Guest editorial: Security and privacy of P2P networks in emerging smart city. Peer-to-Peer Networking and Applications.

[CR112] Li J, Zhang W, Dabra V, Choo K, -. R, Kumari S, Hogrefe D (2019). AEP-PPA: An anonymous, efficient and provably-secure privacy-preserving authentication protocol for mobile services in smart cities. Journal of Network and Computer Applications.

[CR113] Liao, W., Du, W., Salinas, S., & Li, P. (2017). Efficient privacy-preserving outsourcing of large-scale convex separable programming for smart cities. Paper presented at the Proceedings – 18th IEEE International Conference on High Performance Computing and Communications, 14th IEEE International Conference on Smart City and 2nd IEEE International Conference on Data Science and Systems, HPCC/SmartCity/DSS 2016, 1349–1356. 10.1109/HPCC-SmartCity-DSS.2016.0191.

[CR114] Liu JK, Choo K, -. R, Huang X, Au MH (2017). Special issue on security and privacy for smart cities. Personal and Ubiquitous Computing.

[CR115] Lom M, Pribyl O (2020). Smart city model based on systems theory. International Journal of Information Management.

[CR116] Luo X, Ren Y, Hu J, Wu Q, Lou J (2017). Privacy-preserving identity-based file sharing in smart city. Personal and Ubiquitous Computing.

[CR117] Mamonov, S., & Koufaris, M. (2020). Fulfilment of higher-order psychological needs through technology: The case of smart thermostats. *International Journal of Information Management*. 1016/j.ijinfomgt.2020.102091.

[CR118] Manfreda A, Ljubi K, Groznik A (2019). Autonomous vehicles in the smart city era: An empirical study of adoption factors important for millennials. International Journal of Information Management.

[CR119] Maria De Fuentes, J., Gonzalez-Manzano, L., Solanas, A., & Veseli, F. (2018). Attribute-based credentials for privacy-aware smart health services in IoT-based smart cities. *Computer, 51*(7), 44–53. 10.1109/MC.2018.3011042.

[CR120] Mazhelis, O., Hämäläinen, A., Asp, T., & Tyrväinen, P. (2016). Towards enabling privacy preserving smart city apps. Paper presented at the IEEE 2nd International Smart Cities Conference: Improving the Citizens Quality of Life, ISC2 2016 - Proceedings, 10.1109/ISC2.2016.07580755.

[CR121] Mo, Y., Garone, E., Casavola, A., & Sinopoli, B. (2010). False data injection attacks against state estimation in wireless sensor networks. In 49th IEEE Conference on Decision and Control (CDC) (pp. 5967–5972).

[CR122] Moher D, Liberati A, Tetzlaff J, Altman DG (2009). Preferred reporting items for systematic reviews and meta-analyses: the PRISMA statement. Annals of Internal Medicine.

[CR123] Montoya FG, García-Cruz A, Montoya MG, Manzano-Agugliaro F (2016). Power quality techniques research worldwide: A review. Renewable and Sustainable Energy Reviews.

[CR124] Monzon, A. (2015). Smart cities concept and challenges: Bases for the assessment of smart city projects. In *2015 international conference on smart cities and green ICT systems (SMARTGREENS)* (pp. 1–11).

[CR125] Mora, O. B., Rivera, R., Larios, V. M., Beltran-Ramirez, J. R., Maciel, R., & Ochoa, A. (2019). A use case in cybersecurity based in blockchain to deal with the security and privacy of citizens and smart cities cyberinfrastructures. Paper presented at the 2018 IEEE International Smart Cities Conference, ISC2 2018, 10.1109/ISC2.2018.8656694.

[CR126] Moustaka V, Theodosiou Z, Vakali A, Kounoudes A, Anthopoulos LG (2019). Εnhancing social networking in smart cities: Privacy and security borderlines. Technological Forecasting and Social Change.

[CR127] Nam, T., & Pardo, T. A. (2011). Smart city as urban innovation: Focusing on management, policy, and context. In *Proceedings of the 5th international conference on theory and practice of electronic governance* (pp. 185–194).

[CR128] Noh, J., & Kwon, H. (2019). A study on smart city security policy based on blockchain in 5G age. Paper presented at the 2019 International Conference on Platform Technology and Service, PlatCon 2019 - Proceedings, 10.1109/PlatCon.2019.8669406.

[CR129] Oliveira, Á, & Campolargo, M. (2015). From smart cities to human smart cities. In *2015 48th Hawaii International Conference on System Sciences* (pp. 2336–2344). IEEE.

[CR130] Papagiannidis S, Marikyan D (2020). Smart offices: A productivity and well-being perspective. International Journal of Information Management.

[CR131] Pare G, Trudel MC, Jaana M, Kitsiou S (2015). Synthesizing information systems knowledge: A typology of literature reviews. Information & Management.

[CR132] Patsakis C, Laird P, Clear M, Bouroche M, Solanas A (2015). Interoperable privacy-aware E-participation within smart cities. Computer.

[CR133] Pérez-Martínez, P. A., Martínez-Ballesté, A., & Solanas, A. (2013). Privacy in smart cities: A case study of smart public parking. Paper presented at the PECCS 2013 - Proceedings of the 3rd International Conference on Pervasive Embedded Computing and Communication Systems, 55–59.

[CR134] Peris-Ortiz, M., Bennett, D. R., Yábar, D. Pérez-Bustamante (2016). *Sustainable Smart Cities: Creating Spaces for Technological, Social and Business Development*. Springer. ISBN 9783319408958.

[CR135] Peters, F., Hanvey, S., Veluru, S., Mady, A. E., Boubekeur, M., & Nuseibeh, B. (2019). Generating privacy zones in smart cities. Paper presented at the 2018 IEEE International Smart Cities Conference, ISC2 2018, 10.1109/ISC2.2018.8656830.

[CR136] Praharaj S, Han JH, Hawken S (2018). Urban innovation through policy integration: critical perspectives from 100 smart cities mission in India. City, culture and society.

[CR137] Ramos, L. F. M., & Silva, J. M. C. (2019). Privacy and data protection concerns regarding the use of blockchains in smart cities. Paper presented at the ACM International Conference Proceeding Series,, Part F148155 342–347. 10.1145/3326365.3326410.

[CR138] Rana NP, Luthra S, Mangla SK, Islam R, Roderick S, Dwivedi YK (2019). Barriers to the development of smart cities in Indian context. Information Systems Frontiers.

[CR139] Rana NP, Dwivedi YK (2015). Citizen’s adoption of an e-government system: Validating extended social cognitive theory (SCT). Government Information Quarterly.

[CR140] Rana NP, Dwivedi YK, Williams MD (2013). Evaluating the Validity of IS Success Models for E-Government Research: An Empirical Test and Integrated Model. International Journal of Electronic Government Research.

[CR141] Rana NP, Dwivedi YK, Williams MD (2013). E-government adoption research: An analysis of the employee’s perspective. International Journal of Business Information Systems.

[CR142] Rana NP, Dwivedi YK, Williams MD (2013). Evaluating Alternative Theoretical Models for Examining Citizen Centric Adoption of e-Government. Transforming Government: People, Process, and Policy.

[CR143] Rana NP, Dwivedi YK, Williams MD, Lal B (2015). Examining the Success of the Online Public Grievance Redressal Systems: An Extension of the IS Success Model. Information Systems Management.

[CR144] Rana NP, Dwivedi YK, Williams MD, Piercy NC (2015). An Extended DeLone and McLean’s Information System (IS) Model for Examining Success of Online Public Grievance Redressal System in Indian Context. International Journal of Indian Culture and Business Management.

[CR145] Rana NP, Dwivedi YK, Williams MD, Weerakkody V (2015). Investigating Success of an E-Government Initiative: Validation of an Integrated IS Success Model. Information Systems Frontiers.

[CR146] Rana NP, Dwivedi YK, Williams MD, Weerakkody V (2016). Adoption of Online Public Grievance Redressal System in India: Toward Developing a Unified View. Computers in Human Behavior.

[CR147] Rana NP, Williams MD, Dwivedi YK (2013). Analysing Challenges, Barriers and CSFs of E-Government Adoption Research. Transforming Government: People, Process, and Policy.

[CR148] Rohokale, V., & Prasad, R. (2017). Role and importance of the cyber security for developing smart cities in india. Breakthroughs in smart city implementation (pp. 125–146).

[CR149] Rana, N.P., Dwivedi, Y.K., Lal, B., Williams, M.D., and Clement, M. (2017). Citizens’ Adoption of an Electronic Government System: Toward a Unified View. *Information Systems Frontiers, 19*(3), 549–568.

[CR150] Roldan, L. R., Trujillo, A. E., Miyatake, M. N., & Chano, J. (2019). Color watermarking based on DCT and YCbCr color space for privacy preservation in smart cities. Paper presented at the ACM International Conference Proceeding Series, Part F147955 119–123. 10.1145/3316551.3316556.

[CR151] Sanduleac, M., Eremia, M., Toma, L., Alacreu, L., Pons, L., Cresta, M., & Paulucci, M. (2016). Energy ecosystem in smart cities-privacy and security solutions for citizen’s engagement in a multi-stream environment. Paper presented at the IEEE 2nd International Smart Cities Conference: Improving the Citizens Quality of Life, ISC2 2016 - Proceedings, 10.1109/ISC2.2016.07580739.

[CR152] Scuotto V, Ferraris A, Bresciani S (2016). Internet of Things: Applications and challenges in smart cities: a case study of IBM smart city projects. Business Process Management Journal.

[CR153] Sen, M., Dutt, A., Agarwal, S., & Nath, A. (2013). Issues of privacy and security in the role of software in smart cities. Paper presented at the Proceedings – 2013 International Conference on Communication Systems and Network Technologies, CSNT 2013, 518–523. 10.1109/CSNT.2013.113.

[CR154] Shen J, Liu D, Liu Q, He D, Sun X (2017). An enhanced cloud data storage auditing protocol providing strong security and efficiency for smart city. Journal of Information Science and Engineering.

[CR155] Silva BN, Khan M, Han K (2018). Towards sustainable smart cities: A review of trends, architectures, components, and open challenges in smart cities. Sustainable Cities and Society.

[CR156] Simonofski A, Vallé T, Serral E, Wautelet Y (2019). Investigating context factors in citizen participation strategies: A comparative analysis of Swedish and Belgian smart cities. International Journal of Information Management.

[CR157] Singh, P., Dwivedi, Y. K., Kahlon, K. S., Sawhney, R. S., Alalwan, A. A., & Rana, N. P. (2019). Smart monitoring and controlling of government policies using social media and cloud computing. Information Systems Frontiers, 1–23. 10.1007/s10796-019-09916-y.

[CR158] Slade EL, Williams MD, Dwivedi YK (2014). Devising a research model to examine adoption of mobile payments: An extension of UTAUT2. The Marketing Review.

[CR159] Slade, E. L., Williams, M. D., & Dwivedi, Y. K. (2013). Extending UTAUT2 To Explore Consumer Adoption of Mobile Payments. UKAIS 36.

[CR160] Song W, Hu B, Zhao X (2017). Optimizing LWE-based FHE for better security and privacy protection of smart city. Journal of Information Science and Engineering.

[CR161] Sookhak M, Gani A, Khan MK (2015). Dynamic remote data auditing for securing big data storage in cloud computing. Information Sciences.

[CR162] Srivastava, S., Bisht, A., & Narayan, N. (2017). Safety and security in smart cities using artificial intelligence - A review. Paper presented at the Proceedings of the 7th International Conference Confluence 2017 on Cloud Computing, Data Science and Engineering, 130–133. 10.1109/CONFLUENCE.2017.7943136.

[CR163] Statista (2019). https://www.statista.com/statistics/884092/worldwide-spending-smart-city-initiatives/, accessed April 1 2019.

[CR164] Stromire, G., & Potoczny-Jones, I. (2018). Empowering smart cities with strong cryptography for data privacy. Paper presented at the Proceedings of the 1st ACM/EIGSCC Symposium on Smart Cities and Communities, SCC 2018, 10.1145/3236461.3241975.

[CR165] Sucasas V, Mantas G, Althunibat S, Oliveira L, Antonopoulos A, Otung I, Rodriguez J (2018). A privacy-enhanced OAuth 2.0 based protocol for smart city mobile applications. Computers and Security.

[CR166] Sucasas, V., Mantas, G., Radwan, A., & Rodriguez, J. (2016). An OAuth2-based protocol with strong user privacy preservation for smart city mobile e-health apps. Paper presented at the 2016 IEEE International Conference on Communications, ICC 2016, 10.1109/ICC.2016.7511598.

[CR167] Tamilmani, K., Rana, N. P., & Dwivedi, Y. K. (2020). Consumer acceptance and use of information technology: A meta-analytic evaluation of UTAUT2. Information Systems Frontiers, 1–19. 10.1007/s10796-020-10007-6.

[CR168] Techatassanasoontorn, A. A., & Suo, S. (2010). Exploring risks in smart city infrastructure projects: Municipal broadband initiatives. Paper presented at the PACIS 2010–14th Pacific Asia Conference on Information Systems, 13–24.

[CR169] ten Berg, K., Spil, T. A. M., & Effing, R. (2019). The privacy paradox of utilizing the internet of things and wi-fi tracking in smart cities 10.1007/978-3-030-20671-0_25.

[CR170] Townsend A (2013). Smart Cities: Big data, civic hackers, and the quest for a new utopia.

[CR171] Van den Bergh, J., & Viaene, S. (2015). Key challenges for the smart city: Turning ambition into reality. In *2015 48th Hawaii International Conference on System Sciences* (pp. 2385–2394). IEEE.

[CR172] Van Heek, J., Arning, K., & Ziefle, M. (2016). How fear of crime affects needs for privacy & safety: Acceptance of surveillance technologies in smart cities. Paper presented at the SMARTGREENS 2016 - Proceedings of the 5th International Conference on Smart Cities and Green ICT Systems, 32–43.

[CR173] Van Zoonen L (2016). Privacy concerns in smart cities. Government Information Quarterly.

[CR174] Vattapparamban, E., Güvenç, I., Yurekli, A. I., Akkaya, K., & Uluaǧaç, S. (2016). Drones for smart cities: Issues in cybersecurity, privacy, and public safety. Paper presented at the 2016 International Wireless Communications and Mobile Computing Conference, IWCMC 2016, 216–221. 10.1109/IWCMC.2016.7577060.

[CR175] Velasquez, W., Munoz-Arcentales, A., Yanez, W., Salvachua, J. (2018). Resilient smart cities: An approach of damaged cities by natural risks. Paper presented at the 2018 IEEE 8th Annual Computing and Workshop, C., & Conference, C. C. W. C. 2018, 2018-January 591–597. 10.1109/CCWC.2018.8301649.

[CR176] Vitunskaite M, He Y, Brandstetter T, Janicke H (2019). Smart cities and cyber security: Are we there yet? A comparative study on the role of standards, third party risk management and security ownership. Computers and Security.

[CR177] Wachter S (2018). Normative challenges of identification in the Internet of Things: Privacy, profiling, discrimination, and the GDPR. Computer law & security review.

[CR178] Waedt, K., Ciriello, A., Parekh, M., & Bajramovic, E. (2016). Automatic assets identification for smart cities: Prerequisites for cybersecurity risk assessments. Paper presented at the IEEE 2nd International Smart Cities Conference: Improving the Citizens Quality of Life, ISC2 2016 - Proceedings, 10.1109/ISC2.2016.7580812.

[CR179] Walravens N (2015). Mobile city applications for Brussels citizens: Smart City trends, challenges and a reality check. Telematics and Informatics.

[CR180] Wang, L., Jing, C., & Zhou, P. (2012). Security structure study of city management platform based on cloud computing under the conception of smart city. Paper presented at the Proceedings – 2012 4th International Conference on Multimedia and Security, MINES 2012, 91–94. 10.1109/MINES.2012.255.

[CR181] Weerakkody V, Dwivedi YK, Kurunananda A (2009). Implementing e-government in Sri Lanka: Lessons from the UK. Information Technology for Development.

[CR182] Weerakkody, V., Dwivedi, Y. K., Williams, M., Brooks, L., & Mwange, A. (2007). E-government implementation in Zambia: contributing factors. *AMCIS 2007 Proceedings*, 323. Available at: http://aisel.aisnet.org/amcis2007/323.

[CR183] Weerakkody V, Irani Z, Kapoor K, Sivarajah U, Dwivedi YK (2017). Open data and its usability: an empirical view from the Citizen’s perspective. Information Systems Frontiers.

[CR184] Wibowo, S. (2018). Enriching digital government readiness indicators of RKCI assessment with advance https assessment method to promote cyber security awareness among smart cities in indonesia. Paper presented at the Proceeding – 2018 International Conference on ICT for Smart Society: Innovation Toward Smart Society and Society 5.0, ICISS 2018, 10.1109/ICTSS.2018.8549974.

[CR185] Witti, M., & Konstantas, D. (2018). A secure and privacy-preserving internet of things framework for smart city. Paper presented at the ACM International Conference Proceeding Series, 145–150. 10.1145/3301551.3301607.

[CR186] Xiao J, Wang Z, Chen Y, Liao L, Xiao J, Zhan G, Hu R (2017). A sensitive object-oriented approach to big surveillance data compression for social security applications in smart cities. Software - Practice and Experience.

[CR187] Xie J, Tang H, Huang T, Yu FR, Xie R, Liu J, Liu Y (2019). A survey of blockchain technology applied to smart cities: Research issues and challenges. IEEE Communications Surveys & Tutorials.

[CR188] Xie Q, Hwang L (2019). Security enhancement of an anonymous roaming authentication scheme with two-factor security in smart city. Neurocomputing.

[CR189] Yahia NB, Eljaoued W, Saoud NBB, Colomo-Palacios R (2019). Towards sustainable collaborative networks for smart cities co-governance. International Journal of Information Management.

[CR190] Yang F, Xu J (2018). Privacy concerns in china’s smart city campaign: The deficit of china’s cybersecurity law. Asia and the Pacific Policy Studies.

[CR191] Yeh H (2017). The effects of successful ICT-based smart city services: From citizens’ perspectives. Government Information Quarterly.

[CR192] Yilei C, Leyou Z (2019). Privacy preserving ciphertext-policy attribute-based broadcast encryption in smart city. Journal of China Universities of Posts and Telecommunications.

[CR193] Yu W, Xu C (2018). Developing Smart Cities in China: An Empirical Analysis. International Journal of Public Administration in the Digital Age (IJPADA).

[CR194] Zang L, Yu Y, Xue L, Li Y, Ding Y, Tao X (2017). Improved dynamic remote data auditing protocol for smart city security. Personal and Ubiquitous Computing.

[CR195] Zhu Y, Zuo J (2015). Research on security construction of smart city. International Journal of Smart Home.

